# Spatio-temporal modelling and prediction of *Anopheles* mosquito abundance in Tanga and Unguja, Tanzania: climatic drivers and insights for malaria early warning and vector control strategies

**DOI:** 10.1186/s12936-026-05798-z

**Published:** 2026-02-20

**Authors:** Lembris Laanyuni Njotto, Neema B. Kulaya, Yahya A. Derua, Bernard B. Malongo, Filbert Francis, Karin L. Schiøler, Helle Hansson, Christian W. Wang, Fatma Saleh, Vito Baraka, Michael Alifrangis, Tiem van der Deure, Wilfred Senyoni, Ottmar Cronie, Anna-Sofie Stensgaard

**Affiliations:** 1 College of Information and Communication Technologies, University of Dar es Salaam, (CoICT - UDSM), Dar es Salaam, Tanzania; 2 Department of Mathematics and ICT, College of Business Education, Dar es Salaam, Tanzania; 3 KCMC University, Moshi, Tanzania; 4 National Institute for Medical Research, Amani Research Centre, Muheza, Tanzania; 5 National Institute for Medical Research, Tanga Research Centre, Tanga, Tanzania; 6 Global Health Section, Department of Public Health, University of Copenhagen, Copenhagen, Denmark; 7 Centre for Translational Medicine and Parasitology, Department of Immunology and Microbiology, University of Copenhagen, Copenhagen, Denmark; 8 Department of Infectious Diseases, Copenhagen University Hospital, Copenhagen, Denmark; 9 Department of Allied Health Sciences, School of Health and Medical Sciences, The State University of Zanzibar, Zanzibar, Tanzania; 10 Department of Mathematical Sciences, Chalmers University of Technology and University of Gothenburg, Gothenburg, Sweden; 11 Department of Veterinary and Animal Sciences, University of Copenhagen, Frederiksberg, Denmark

**Keywords:** *Anopheles*, Climate, Forecasting, GAMM, Malaria, Tanga, Tanzania, Unguja, Zanzibar

## Abstract

**Background:**

*Anopheles* mosquitoes, vectors of human malaria, are highly sensitive to environmental change. As climate alters temperature and precipitation patterns, mosquito populations may shift in sibling species composition, location and timing, altering transmission dynamics. Understanding these patterns is key for malaria control. This study explores links between meteorological factors and *Anopheles* abundance across a diversity of sites in Tanga and Unguja, Tanzania, to predict mosquito peaks and support the development of early warning systems for malaria outbreaks.

**Methods:**

Adult *Anopheles* mosquitoes were sampled monthly from September/October 2021 to December/September 2023 across 11 sites in Tanga and 4 shehias in Unguja. Spatio-temporal Generalized Additive Mixed Effects Models (GAMMs) were employed to assess the influence of meteorological factors on *Anopheles* abundance. Models were built and validated using mosquito counts alongside climate covariates obtained from Copernicus ERA5-Land and NASA’s POWER platforms.

**Results:**

A total of 4312 adult *Anopheles* mosquitoes were sampled in Tanga and 1450 in Unguja. The GAMM revealed region-specific climatic drivers. In Tanga, *Anopheles* abundance increased with higher maximum temperatures but declined with higher minimum temperatures. In Unguja, abundance exhibited a non-linear relationship with mean temperature, peaking below 27.5 °C and decreasing thereafter. Precipitation in Tanga positively influenced *Anopheles* abundance both concurrently and with a two-month lag, whereas in Unguja only the two-month lag effect was significant. Relative humidity exhibited a non-linear effect in both regions, with higher humidity associated with increased abundance. The GAMMs demonstrated strong predictive performance as evidenced by low MAE and RMSE, Theil’s U < 1, and correlation exceeding 0.8 between observed and predicted values. Importantly, the models accurately forecasted *Anopheles* abundance peaks in Unguja in November 2023, preceding the reported malaria surge in Zanzibar in late 2023 and early 2024, highlighting its potential as a proxy for malaria risk and a scalable early warning system to support proactive targeted vector control.

**Conclusion:**

The study highlights the importance of integrating meteorological variability into mosquito surveillance and control. The spatio-temporal GAMM captured weather-driven mosquito dynamics and predicted surges in *Anopheles* abundance prior to the Zanzibar malaria outbreak in late 2023. These insights can guide targeted interventions across diverse eco-climatic regions, enhancing malaria vector control.

**Supplementary Information:**

The online version contains supplementary material available at 10.1186/s12936-026-05798-z.

## Introduction

Mosquitoes are vectors of numerous parasitic and viral infections, posing a substantial burden on global health, particularly in the tropical and subtropical regions where transmission is most intense [1]. Among these, malaria, caused by *Plasmodium* parasites, remains one of the deadliest [2]. According to the 2025 World Health Organization (WHO) malaria report, an estimated 283 million malaria cases and approximately 610,000 deaths occurred globally in 2024 [3], an increase of 12,000 deaths compared with 2023 [4]. This corresponds to an incidence rate of 64.0 cases per 1000 people at risk [3], representing a 2% increase from the 62.7 cases per 1000 reported in 2023 [4]. Africa continues to bear the overwhelming burden of the disease, accounting for 94% of global cases and 95% of deaths. In 2024, 4.3% of all malaria deaths occurred in the United Republic of Tanzania, making it the fourth most affected country in the world [3]. While the country as a whole continues to bear a significant malaria burden, Zanzibar—a Tanzanian semi-autonomous archipelago off the coast of East Africa—has maintained a malaria prevalence below 1% for over a decade [5]. However, a recent surge in cases on Unguja Island, the larger of Zanzibar’s two main islands, has raised serious concern, as nearly 24,000 cases were reported between November 2023 and March 2024, an unprecedented increase compared to previous years [6, 7].

Human malaria is transmitted by infected female mosquitoes of the genus *Anopheles.* In Africa, two groups of *Anopheles* are primarily responsible for transmitting malaria parasites: the *Anopheles gambiae* complex and the *Anopheles funestus* group [8, 9]. As mosquito density is a critical driver of transmission, several vector control measures have been implemented to reduce the mosquito populations and ultimately lessen the burden of malaria in affected communities. These include the use of insecticide-treated bed nets (ITNs) and indoor residual spraying (IRS) [10]. However, the effectiveness of vector control is increasingly challenged by several factors including rising insecticide resistance [8, 11], and shifts in vector composition and behaviour, which for instance have led to outdoor biting [12]. In addition, the growing impact of climate change is altering environmental conditions essential for mosquito survival and development [13].

Climate and climate change play a fundamental role in shaping mosquito populations by directly influencing the environmental conditions essential for their survival and reproduction. Mosquito distribution and population dynamics are closely tied to microclimates, with rainfall, temperature, and humidity serving as key regulatory factors [14–16]. Rainfall is essential for mosquito breeding, as stagnant water provides sites for egg-laying and larval development. Moderate rainfall replenishes breeding sites like ponds and puddles, fostering population growth, whereas insufficient rainfall can dry up these habitats, and excessive rainfall can wash away eggs and larvae [16, 17]. Temperature also plays a critical role with moderate and warm conditions accelerate larval development, metabolism, and egg production, while extreme temperatures hinder these processes, reducing survival rates [18, 19]. Humidity further influences mosquito populations, affecting egg production, larval development, and adult survival. High humidity enhances mosquito activity and longevity, whereas low humidity can desiccate eggs and reduce adult survival [20–22].

This relationship between climate and vector mosquito population distribution and dynamics, can be used to develop predictions models that can help identify and monitor emerging high-risk areas, which ultimately can help strengthen malaria control efforts and attempt to mitigate possible impact of climate changes on vector infestation [23, 24]. However, the impact of weather and climate on mosquito populations is complex and often nonlinear [25, 26]. Furthermore, the relationship between climatic factors and mosquito dynamics is context-specific and may vary between geographical areas. While various modelling approaches exist, many face limitations such as oversimplifying relationships due to their assumptions (linearity) or lacking interpretability (complexity of the model), which can hinder effective ecological understanding and public health application [27, 28].

This study aims to explore the relationship between climatic factors and *Anopheles* mosquito abundance using spatio-temporal Generalized Additive Mixed Effect Models (GAMMs), that can represent nonlinear relationships [29, 30]. Specifically, we utilized GAMMs to analyse the effect of meteorological variables on monthly mosquito count data collected from multiple sampling sites across the Tanga and Unguja regions of the United Republic of Tanzania (between September 2021 and December 2023). Besides providing deeper insight into how weather patterns influence mosquito population fluctuations in these areas, the GAMMs can be used to forecast *Anopheles* mosquito abundance, which in turn may serve as a proxy for potential malaria outbreaks and inform the timely design and more effective allocation of limited resources for targeted interventions under climate variability.

## Methodology

### Study area

**Fig. 1:**
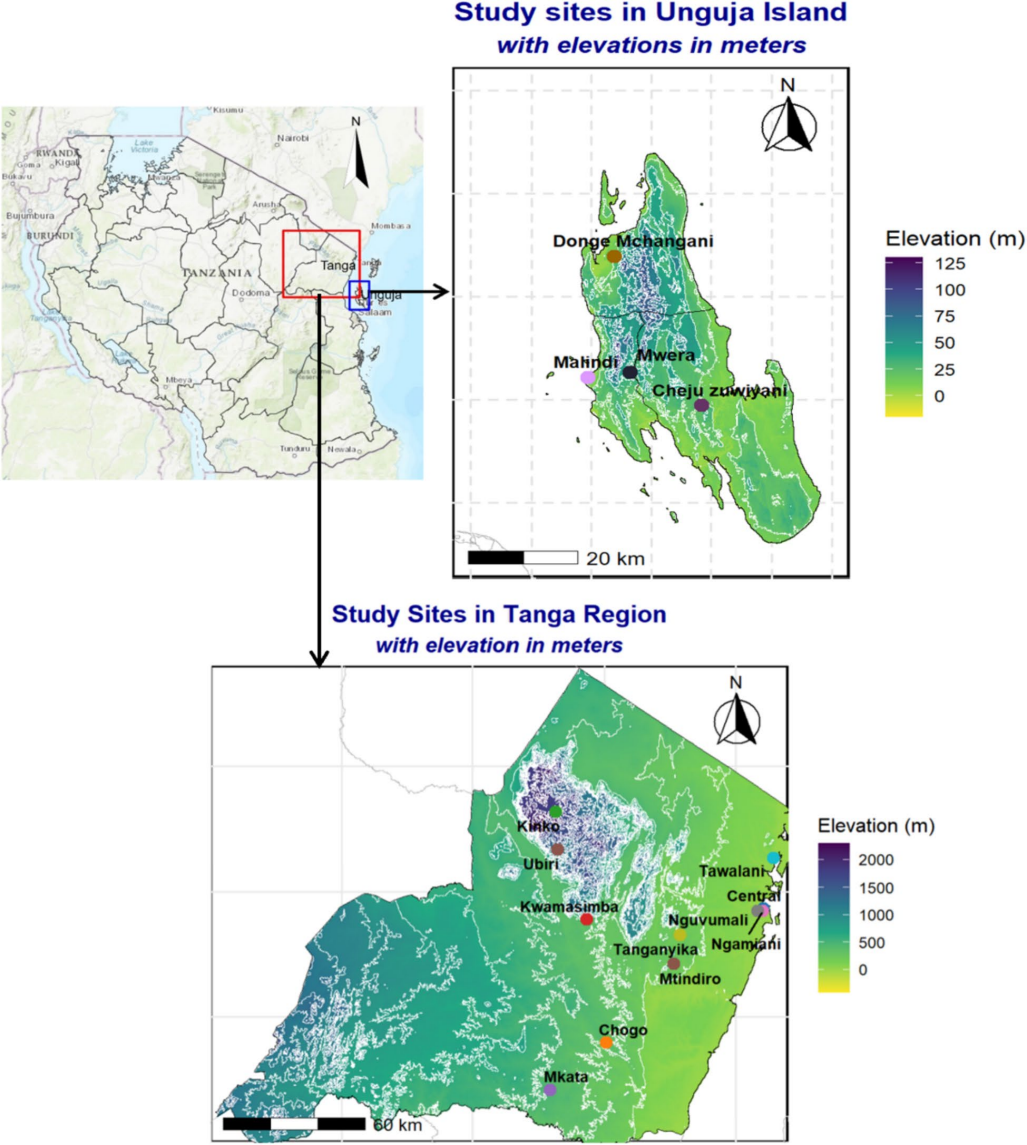
The Tanzania map showing the study sites of Tanga and Unguja, with specific locations where monthly mosquito sampling was conducted

The study was conducted in two regions of the United Republic of Tanzania: the Tanga region on the mainland and the Unguja island in Zanzibar (Fig. 1). Tanga is one of Tanzania’s 31 administrative regions, located at a latitude of 5.09°S and a longitude of 39.10°E, and occupying a land area of 26,677 km^2^ [31]. According to the 2022 Population and Housing Census, Tanga has a population of 2.6 million [32]. The region ranges from coastal plains to the Usambara Mountains, expanding to about 2289 m above the sea level. Tanga experiences a tropical climate, with annual rainfall between 961 and 1885 mm, peaking from March to May and again from October to December. Its diverse landscape includes rainforests in the highlands and humid savannahs along the coast. Administratively, Tanga is divided into 10 districts and 227 wards, with its residents engaged in various economic activities, primarily agriculture, both large-scale and subsistence, alongside fishing, livestock keeping, and employment in formal and informal sectors; major crops include sisal, coconuts, maize, oranges, and vegetables. Mosquito-borne diseases such as malaria, lymphatic filariasis (LF), and arboviruses are common in Tanga, as it is in many other regions of the country [33, 34]. Malaria is the most prevalent of these diseases, with transmission occurring even in high-altitude areas [35, 36]. Malaria transmission persists throughout most of the year, with two seasonal peaks, following the heavy rains from March to May and during the short rainy season between October and December [37]. The region harbours a mixed population of malaria vectors, primarily *An. gambiae* complex and the *An. funestus* complex [12].

Part of the study was conducted in Unguja, one of the two main islands of the Zanzibar archipelago off the coast of mainland Tanzania. Of Zanzibar’s total population of 1889,773, nearly 70% reside on Unguja, while the remainder live on Pemba [32]. Administratively, Unguja is divided into three regions, seven districts, and 259 shehias-the smallest local units [31]. The island experiences a tropical climate with two rainy seasons (October–December and March–May). The major economic activities in Unguja include subsistence farming, fishing, livestock keeping, and tourism, with key crops including spices, rice, bananas, fruits, and vegetables. Malaria remains the most prevalent vector-borne disease in Unguja Island, with *An. gambiae* complex being the primary vectors [38].

In both Tanga (mainland Tanzania) and Unguja (Zanzibar), a two-stage preferential sampling scheme was applied to select sites that captured diverse demographic, eco-climatic, and eco-geographical conditions for mosquitoes sampling. In the first stage, 15 study locations were purposively selected (11 sites in Tanga and 4 shehias in Unguja) ensuring that sites within each region were at least 10 km apart to minimize the likelihood of pseudo-replication of sampled data. In Tanga, the 11 sites were distributed across six districts, ranging from the coastal areas of Tanga City to the elevated terrain of the Usambara Mountains, and included six rural sites (Mtindiro, Chogo, Tawalani, Kwamasimba, Ubiri, and Kinko), two semi-urban sites (Mkata Mashariki and Tanganyika), and three urban sites (Central, Nguvumali, and Ngamiani Kaskazini) (Fig. 1). In Unguja, four shehias were selected to represent urban (Malindi), semi-urban (Mwera), and rural (Cheju Zuwiyani and Donge Mchangani) settings (Fig. 1). In the second stage, 10 households per site or shehia were purposively chosen in close proximity to known mosquito breeding habitats to maximize the likelihood of capturing *Anopheles* mosquitoes. Monthly collections were conducted both indoors and outdoors over two years period (September 2021–December 2023 in Tanga, and October 2021 to September 2023 in Unguja) covering both wet and dry seasons. Additionally, out-of-sample entomological data from Unguja were obtained from the Zanzibar Malaria Elimination Programme (ZAMEP) to supplement the primary dataset.

### Adult *Anopheles* mosquito sampling

The mosquito sampling process was carefully designed to capture both seasonal and behavioural variations in *Anopheles* populations across Tanga and Unguja. Field teams visited selected households each month, ensuring that data collection spanned both the dry and rainy seasons. This systematic approach allowed researchers to monitor fluctuations in mosquito abundance and behaviour under different environmental conditions. A standardized protocol was followed to sample mosquito vectors both indoors and outdoors, providing insights into the spatiotemporal dynamics of adult mosquitoes. To account for diverse mosquito behaviours and habitats, multiple sampling methods were employed. Resting mosquitoes were collected using Prokopack aspirators between 06:00 and 10:00 h, with 15 min dedicated to indoor sampling and 30 min outdoors per household. Host-seeking mosquitoes were targeted using CDC light traps placed indoors near beds where volunteers slept under bed nets. These traps operated overnight from 18:00 to 07:00 h, attracting mosquitoes that sought human hosts indoors. In parallel, Furvela tent traps were deployed outdoors from 18:00 to 06:00 h to capture nocturnally active host-seeking *Anopheles* mosquitoes. All captured mosquitoes were identified using morphological characteristics, categorized by genus and sex, and documented in their adult form. The data were systematically recorded, and all households were georeferenced to facilitate spatial analysis.

### Meteorological data

Monthly meteorological data for each sampling site in Tanga and each shehia in Unguja were compiled from a combination of satellite-derived and reanalysis-based sources. Near-surface temperature variables, including maximum, minimum, and mean, and relative humidity were extracted from the Copernicus ERA5-Land dataset [39], which offers hourly climate variables at a spatial resolution of approximately 0.1° × 0.1°. As relative humidity is not directly provided in ERA5-Land, it was derived using standard thermodynamic equations based on 2-m air temperature and dew point temperature. Precipitation estimates were sourced from the NASA Prediction of Worldwide Energy Resources (POWER) platform [40], specifically utilizing the Integrated Multi-satellite Retrievals for GPM (IMERG) algorithm [41, 42], also at a resolution of 0.1° × 0.1°. These satellite datasets were cross validated against records from nearby meteorological stations using lines comparisons and correlation analysis, showing good agreement with results presented elsewhere [43]. This validation step was particularly important given the incomplete coverage of local weather stations across the study area. Prior to model development, multicollinearity among meteorological variables was evaluated using Variance Inflation Factor (VIF) analysis [44], applying a threshold of VIF < 10 to ensure each variable contributed unique information and to improve both the precision and interpretability of the results.

### Model development

To explore how climatic variables influence the abundance of *Anopheles* mosquitoes, line plots were utilized as part of an exploratory data assessment. These visualizations offered valuable insights into how mosquito counts varied with environmental factors such as temperature, humidity, and precipitation. Given the geospatial nature of the data, collected from specific locations across various sites and shehias, and shaped by both spatial and temporal dynamics, a modelling approach capable of capturing this complexity is essential. Accordingly, we employed a spatio-temporal Generalized Additive Mixed Effect Models (GAMM), which allowed for flexible, non-linear modelling through smooth functions, while effectively accounting for spatial and temporal dependencies [45, 46].

Let ${Y}_{it}$ denote the count of *Anopheles* mosquitoes observed at location $i$, during time $t$. Given the discrete, count-based, and non-negative nature of the data, a Poisson distribution is assumed for ${Y}_{it}$. The expected count, ${\mu }_{it}$ is associated to the covariates through

1 $$\begin{array}{*{20}c} {{\mathrm{g}}\left( {\mu_{it} } \right) = \beta_{0} + \sum_{{{\mathrm{k}} = 1}}^{{\mathrm{K}}} \beta_{k} (X_{kit} ) + Z_{1} \left( {T_{t} } \right) + Z_{2} \left( {S_{t} } \right),} \\ \end{array}$$

where $g\left(\cdot \right)$ is the canonical log link function for the Poisson distribution with expectation ${\mu }_{i\mathrm{t}}=E\left({Y}_{it}\right)$. Here, ${\beta }_{0}$ is the model intercept, ${X}_{kit}$**,** represents the ${k}^{th}$ climatological or meteorological covariates at location $i$ and time $t$, ${Z}_{1}\left({T}_{t}\right)$ is a smooth function of time, capturing temporal variation such as seasonal or monthly trends, ${Z}_{2}\left({S}_{t}\right)$ is a smooth random effect for site, which accounts for site-specific variability and enables estimation and forecasting at the site level. To account for temporal dependence in the repeated measurements collected within each site, we specified a first‑order autoregressive correlation structure (AR(1)) using the corAR1 class in the GAMM model. This structure models short‑range temporal autocorrelation, meaning that observations taken closer together in time are more strongly correlated than those further apart. Incorporating AR(1) therefore complements the temporal smooth by capturing residual serial dependence that is not explained by seasonal patterns alone, ensuring that both long‑term trends and within‑site temporal correlation are appropriately represented in the final GAMM.

The smooth functions in the model are formulated using regression splines, expressed as linear combinations of basis functions applied to the covariates:

2 $$\beta_{k} (X_{kit} ) = { }\mathop \sum \limits_{j = 1}^{J} \alpha_{j} \psi_{j} \left( {{\mathrm{X}}_{kit} } \right).$$

In this context, ${\psi }_{j}$ denotes the basis functions, while ${\alpha }_{j}$ represent the parameters to be estimated. The number of basis functions, $J$, determines the maximum flexibility in modelling the relationship between ${X}_{kit}$ and $\mathrm{g}\left({\mu }_{it}\right)$; a higher value of $J$ allows the model to capture more complex, non-linear effects. For this study, we employed a Cubic Regression Splines (CRS), where the range of each predictor is partitioned into segments separated by knots. Within each segment, a local cubic polynomial is fitted. To ensure smoothness and continuity at the knots, constraints are imposed on the first and second derivatives of the spline functions [47]. The smoothing parameters controlling the trade-off between fit and smoothness were estimated via Restricted Maximum Likelihood (REML), a method known for its robustness and reduced bias, especially in models with random effects, yielding stable and reliable estimates [46].

### Model fitting and forecasting

In modelling count data, the Poisson distribution is a common starting point, as it assumes that the mean and variance of the response are equal. However, this assumption is often violated in real-world data, which frequently exhibit overdispersion (where the variance exceeds the mean) or excessive number of zeros [48–50]. To handle overdispersion, alternative distribution models such as the negative binomial or quasi-Poisson are typically employed. These models incorporate an additional dispersion parameter that allows the variance to differ from the mean, thereby offering a better fit to data exhibiting higher variability [49, 51]. In scenarios where the dataset contains a high proportion of zeros, zero-inflated models become more appropriate. These models extend the traditional count framework by introducing components to separately model the excess zeros, distinguishing between structural (true) and sampling (random) zeros [50]. To determine the most appropriate model specification, we compared multiple candidate distributions using the Akaike Information Criterion (AIC) and Bayesian Information Criterion (BIC). For models employing a quasi-Poisson family, which lacks a true likelihood function, we instead used the Quasi-Akaike Information Criterion (QAIC) and Generalized Cross-Validation (GCV) score. These metrics quantify the trade-off between model fit and complexity, where lower values indicate a better-fitting model.

Prior to model fitting, the dataset was partitioned into training (80%) and test (20%) sets to facilitate robust evaluation of model performance [52]. For the Tanga sites, the training set comprised data from September 2021 to June 2023, while the test set included observations from July to December 2023. Similarly, for the shehias data in Unguja, training data ranged from October 2021 to May 2023, with the test period covering June to September 2023. To further assess predictive performance and model generalizability, we conducted an additional evaluation using available out-of-sample data, specifically, mosquito counts from October to December 2023, sourced from the Zanzibar Malaria Elimination Programme (ZAMEP). Model forecasts were compared against both the test set derived from the same dataset and this independent, external dataset to evaluate consistency and reliability across samples.

### Model performance

To evaluate the model's predictive performance, we calculated several error metrics: Mean Absolute Error (MAE), which measures the average size of prediction errors; Normalized Root Mean Square Error (NRMSE), which scales the error relative to the data range [53, 54]; the correlation between observed and predicted values, indicating the degree of association; and Theil’s U statistic, which enables a comparative analysis of forecasting methods by weighting larger errors more heavily and benchmarking against a naïve forecast [54]. Lower values of MAE, NRMSE, and Theil’s U, along with higher correlation coefficients, indicate better predictive performance.

All statistical analyses were conducted using R statistical software (version 4.5.0) (CRAN, 2024) using the packages *mgcv* [45, 55].

## Results

### The distribution of *Anopheles* mosquitoes in Tanga

Between September 2021 and December 2023, a total of 4312 *Anopheles* mosquitoes were collected from eleven sites in Tanga (Table 1). Morphological identification revealed two main species, with *An. gambiae* being the dominant one, comprising 82.0% of the total, while *An. funestus* accounted for 18.0%. Female mosquitoes accounted for 93.4% of the total catch, with *An. gambiae* comprising 77.1% and *An. funestus* 16.3%. The majority of these mosquitoes were collected from rural sites (77.5%), with Mtindiro recording the highest proportion (65.7%), followed by the semi-urban site of Mkata Mshariki (19.7%). In Unguja between October 2021 and September 2023, 1450 *Anopheles* mosquitoes were collected from four shehias (Table 1). The species distribution resembled that of Tanga, with *An. gambiae* comprising 95.0% and *An. funestus*; 5.0%. Similarly, female mosquitoes were predominant, comprising 80.6% of the catch, of which 77.3% were *An. gambiae* and 3.3% were *An. funestus*. Consistent with the pattern observed in Tanga, the majority of these mosquitoes were collected from rural areas, with Cheju Zuwiyani recording the highest proportion (73.0%), followed by the semi-urban shehia of Mwera (17.2%). In addition to morphological identification, further molecular characterization of the sibling species was conducted, and those results are presented in a separate manuscript that focuses specifically on the genetic identification of the vector species [56].

**Table 1:** The number of *Anopheles* mosquitoes captured during the study period from eleven sites in Tanga and from four shehias in Unguja, Tanzania

Tanga								
Location	Site		*An. gambiae s.l*		*An. funestus s.l*		Total n (%)	
	Sites	Altitude (m)	Female	Male	Female	Male		
Rural	Mtindiro	330	2127	61	630	16	2834 (65.7)	3342 (77.5)
	Chogo	342	282	19	10	0	311 (7.2)	
	Tawalani	0	74	1	9	21	105 (2.4)	
	Kwamasimba	662	19	0	13	8	40 (0.9)	
	Ubiri	1214	19	1	8	4	32 (0.7)	
	Kinko	1701	13	0	5	2	20 (0.5)	
Semi-urban	Mkata Mashariki	371	713	128	4	3	848 (19.7)	889 (20.6)
	Tanganyika	166	21	0	14	6	41 (0.9)	
Urban	Central	0	50	0	10	15	75 (1.7)	81 (1.9)
	Ngamiani Kaskazini	7	4	0	0	0	4 (0.1)	
	Nguvumali	0	2	0	0	0	2 (0.1)	
Total n (%)			3324 (77.1)	210 (4.9)	703 (16.3)	75 (1.7)	4312	
			3534 (82.0)		778 (18.0)			
Unguja								
Rural	Cheju Zuwiyani	0.2	882	153	8	16	1059 (73.0)	1091 (75.2)
	Donge Mchangani	0.0	27	5	0	0	32 (2.2)	
Semi-urban	Mwera	12.3	133	93	24	0	250 (17.2)	250 (17.2)
Urban	Malindi	0.0	79	5	16	9	109 (7.5)	109 (7.5)
Total n (%)			1121 (77.3)	256 (17.7)	48 (3.3)	25 (1.7)	1450	
			1377 (95.0)		73 (5.0)			

### Monthly *Anopheles* mosquito distribution in Tanga and Unguja

**Fig. 2:**
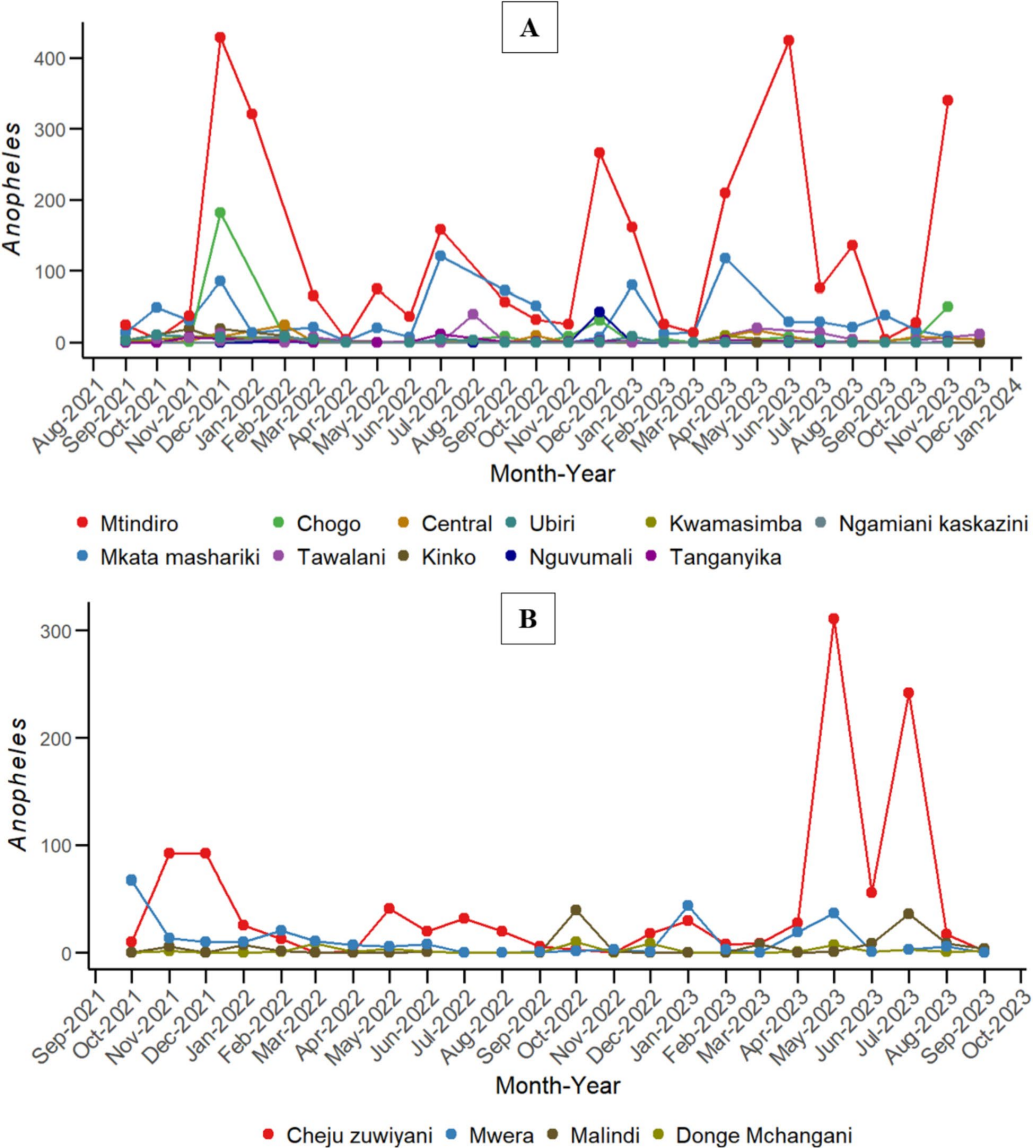
Trends in Anopheles mosquito abundance across various sites in Tanga (**A**) and shehias in Unguja (**B**), Tanzania, observed over the study period

The monthly distribution of *Anopheles* mosquitoes collected from eleven sites in Tanga (September 2021–December 2023) and from four shehias in Unguja (October 2021–September 2023) are depicted in Fig. 2. In Tanga, Mtindiro site had the highest mosquito counts, consistently peaking at multiple points during the study period (Fig. 2A). Significant increases were recorded in December 2021, December 2022, and June 2023, with mosquito counts exceeding 400 in both December 2021 and June 2023. Other sites showed moderate mosquito counts, with occasional peaks that were less pronounced than those observed in Mtindiro. In Unguja, Cheju Zuwiyani consistently recorded the highest mosquito counts among the shehias (Fig. 2B). Notable peaks occurred in November and December 2021, as well as in May, and July 2023, with the highest count exceeding 200 mosquitoes in May and July 2023. Other shehias exhibited moderate fluctuations in *Anopheles* mosquito counts, but none reached the elevated levels observed in Cheju Zuwiyani.

### Temporal relationship between *Anopheles* abundance and climatic factors

Prior to model fitting, we assessed potential multicollinearity among climatic variables. In the Tanga dataset, average temperature showed strong correlations with both maximum and minimum temperatures. In Unguja, similar correlations were observed, along with a strong relationship between maximum and minimum temperatures. The Variance Inflation Factor (VIF) was applied to quantify the degree of multicollinearity. As a result, in Tanga, average temperature was excluded from the model, while maximum and minimum temperatures were retained. Conversely, in Unguja, average temperature was retained, and both maximum and minimum temperatures were excluded, as they exhibited higher VIF values.

**Fig. 3:**
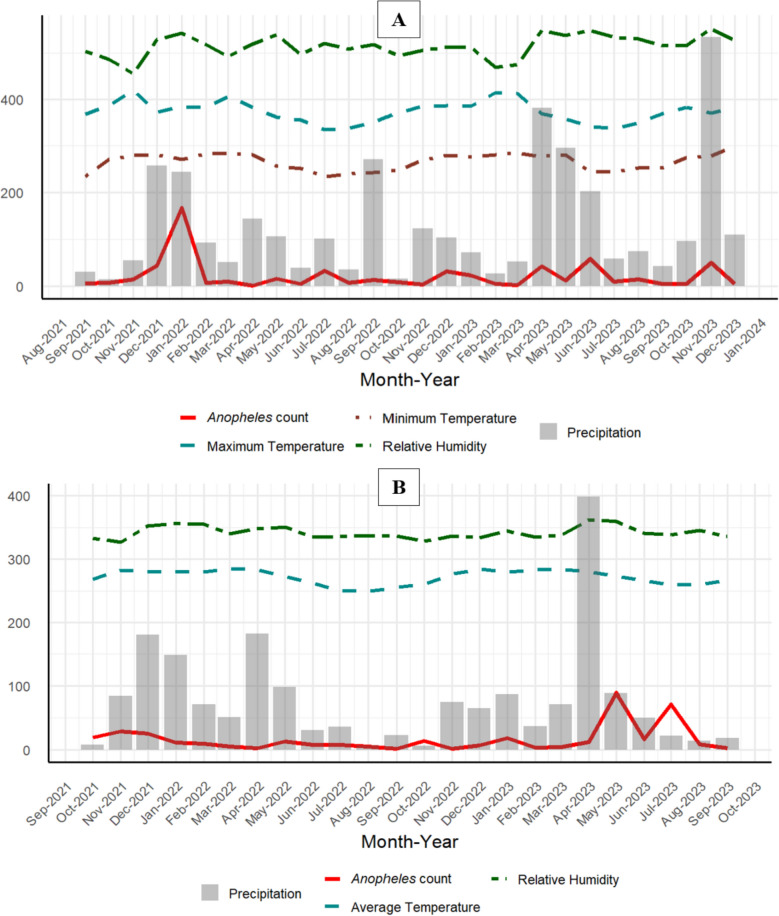
Time series of mean number of *Anopheles* mosquitoes, with scaled climatic variables including precipitation, temperatures (average, minimum, and maximum), and relative humidity in (**A**) Tanga (Sept 2021-Dec 2023) and (**B**) Unguja (Oct 2021-Sept 2023)

The relationships between the average number of *Anopheles* mosquitoes over time and the scaled climatic variables in Tanga and Unguja are presented in both Tanga (Fig. 3A) and Unguja (Fig. 3B). A positive association was observed between mosquito counts and precipitation in both regions, with correlation coefficients of 0.52 in Tanga and 0.16 in Unguja. In Tanga, mosquito abundance peaked in early 2022, coinciding with increased precipitation levels. Similarly, in Unguja, peaks in mosquito counts were recorded in May and July 2023, aligning with increased precipitation during those months or preceding periods. Relative humidity also demonstrated a positive relationship with *Anopheles* abundance (0.61 in Tanga and 0.21 in Unguja). Temperature-related variables showed weaker associations: both maximum and minimum temperatures in Tanga and average temperature in Unguja remained relatively stable over time, resulting in only marginal positive correlations with mosquito abundance. Nonetheless, months with slightly higher temperatures tended to coincide with modest increases in *Anopheles* mosquito counts.

### Model results

Model selection indicated clear differences between the two regions. In Tanga, mosquito abundance was best described using a negative binomial distribution, while in Unguja a zero-inflated Poisson distribution provided the best fit. In Tanga, maximum and minimum temperatures showed linear relationships with *Anopheles* abundance, whereas minimum temperature (lagged one month), relative humidity, and precipitation exhibited non-linear effects. In Unguja, all climatic variables, including average temperature, relative humidity, and precipitation lagged by two months, displayed significant non-linear associations with mosquito abundance. The interaction between average temperature and relative humidity was also significant in Unguja but not in Tanga.

The results of the GAMM are summarized in Table 2. The model's performance evaluation demonstrated that it explained approximately 85.1% of the deviance in mosquito abundance in Tanga, indicating a strong fit and effectively capturing most of the variability in mosquito populations. In Unguja, the model explained 75.9% of the deviance, slightly lower than in Tanga but still reflecting a reasonably good fit. Altitude exhibited a distinct influence on the abundance of *Anopheles* mosquitoes in Tanga, with each one-meter increase in altitude corresponding to a decreased density of *Anopheles* mosquito by a factor of 0.003. Conversely, altitude was as expected, not a significant factor in Unguja and was excluded from the model, likely due to the region’s predominantly flat topography, which offers minimal altitude variation across sites. In Tanga, the linear effects of maximum and minimum temperatures revealed that the incidence rate ratios (IRRs) (exponentiating the estimate) were approximately 1.388 and 0.607, respectively (Table 2). This indicates that for each 1 °C increase in maximum temperature, the abundance of *Anopheles* mosquitoes increased by about 38.8%, while a 1 °C increase in minimum temperature led to a 39.3% decrease in *Anopheles* abundance.

**Table 2:** Model estimates of the effects of weather on the abundance of *Anopheles* mosquitoes in Tanga and Unguja, Tanzania

Linear terms	Tanga			Unguja		
	Estimates	Std	p-value	Estimates	std	p-value
Intercept	4.08	3.18	0.20	1.66	0.66	0.011
Altitude	− 0.003	0.0015	0.035			
Maximum temperature	0.328	0.133	0.015			
Minimum temperature	− 0.499	0.167	0.003			

Smooth terms	Edf	p-value	edf	p-value
s (Average temperature)			1.10	0.014
s (Maximum temperature lag 1)	2.517	0.0074		
s (Relative humidity)	2.308	< 0.0001	3.51	< 0.0001
s (Total precipitation)	0.937	0.0004		
s (Total precipitation Lag 2)	1.698	0.036	1.584	0.0028
ti (Average temperature, relative humidity)			4.725	0.026

Random effects	Edf	p-value	edf	p-value
s (months)	7.713	< 0.0001	9.054	< 0.0001
s (sites/shehias)	8.195	< 0.0001	2.981	< 0.0001

Model statistics	Estimates	Estimates
Deviance explained	85.1%	75.9%

Lag 1 and Lag 2 indicate month-lagged values of the variables by one and two months, respectively, s() refers to a smooth function applied to a continuous variable using a spline, ti() stands for a tensor product interaction term between two variables, edf refers to the estimated degrees of freedom for each smooth term, a higher edf indicates a more complex, flexible function (more “wiggly”), while a lower edf (~ 1) suggests a nearly linear relationship

### Smoothed climatic effects on *Anopheles* abundance

**Fig. 4:**
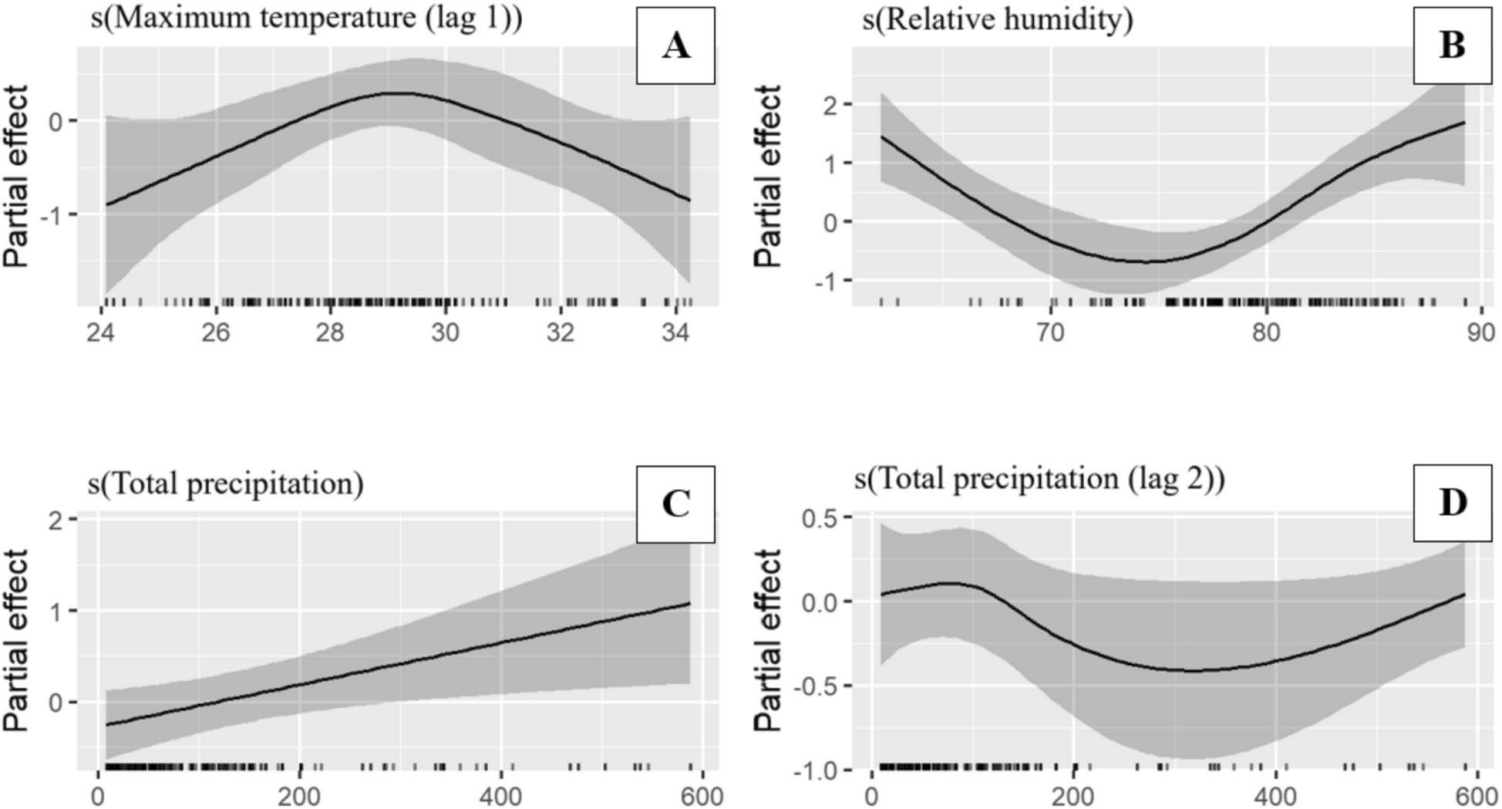
GAMM smoothing curves depicting the partial effects of model covariates on *Anopheles* mosquito abundance in Tanga. Tick marks on the x-axis represent observed data points, while the y-axis shows the partial effect of each variable. Shaded areas denote the 95% confidence intervals

Figure 4 presents the response plots from the fitted model, illustrating the relationship between *Anopheles* mosquito abundance and climatic variables in Tanga. All the analysed non-linear climatic factors showed a significant relationship with mosquito abundance, though the nature of these associations varied. Maximum temperature lagged by one month (Fig. 4A) had minimal effect on mosquito abundance at lower temperature ranges but exhibited a negative effect at temperatures above 30 °C. *Anopheles* abundance decreased as relative humidity increases from low levels, stabilized between 70 and 80%, and began to increase again at humidity levels above 80%. Total precipitation during the month of mosquito collection (Fig. 4C) showed a positive correlation with *Anopheles* abundance, with higher precipitation levels (> 200 mm) leading to increased abundance. Additionally, total precipitation lagged by two months (Fig. 4D) had a negative influence at lower precipitation levels, but a positive trend was observed at higher precipitation levels (> 400 mm), which might indicate that higher precipitation levels provide favourable breeding conditions for mosquitoes after an initial lag period.

**Fig. 5:**
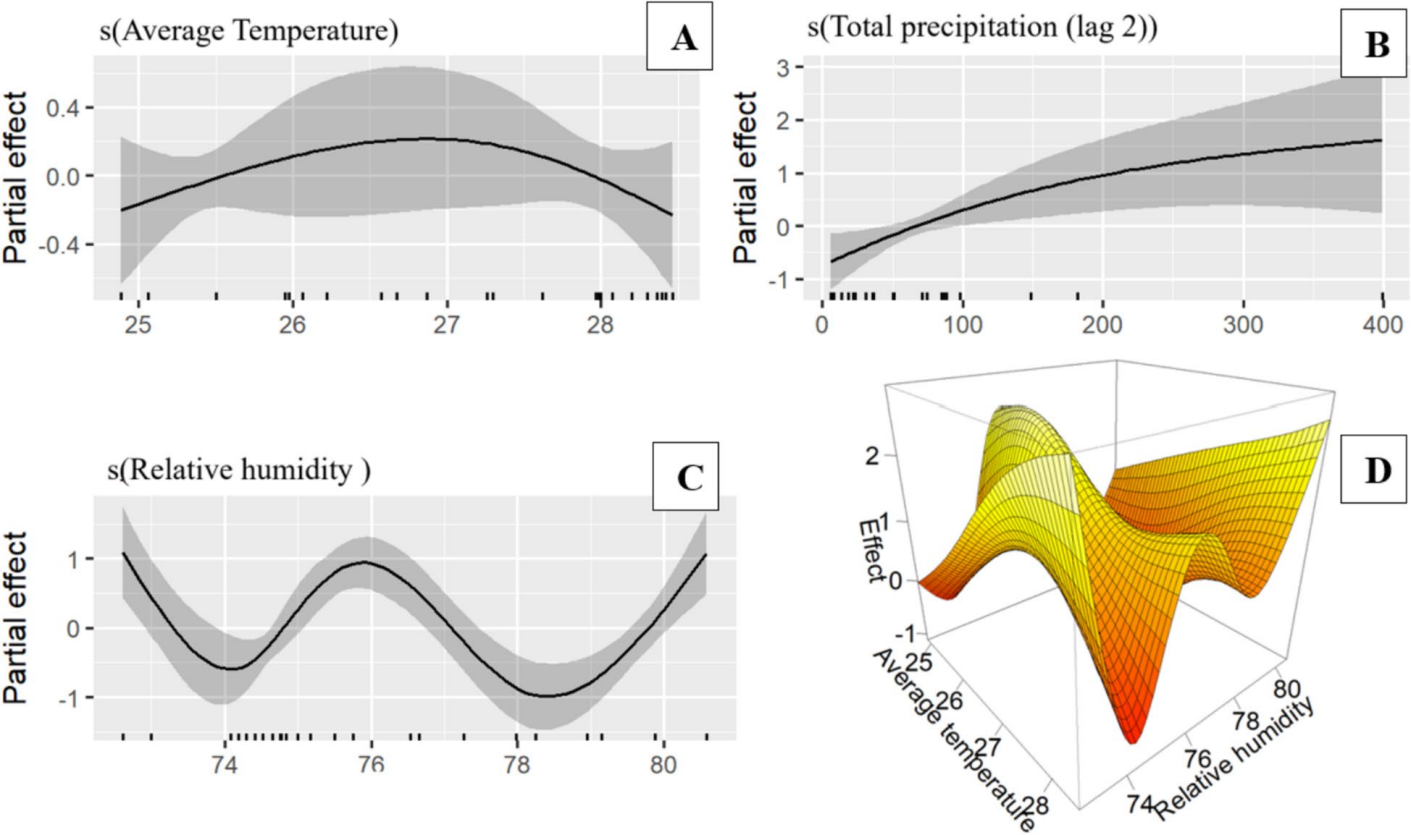
GAMM smoothing curves depicting the partial effects of model covariates on *Anopheles* mosquito abundance in Unguja. Tick marks on the x-axis represent observed data points, while the y-axis shows the partial effect of each variable; temperature (**A**), precipitation (**B**), and relative humidity (**C**). Shaded areas denote the 95% confidence intervals. D illustrates the interaction effects between average temperature and relative humidity on *Anopheles* mosquitoes in Unguja

For Unguja, *Anopheles* mosquito abundance revealed that the number of mosquitoes increased as average temperature increases initially, but when temperatures exceeded 27.5 °C, *Anopheles* abundance declined (Fig. 5A). Precipitation lagged by two months (Fig. 5B) had a positive effect on *Anopheles* mosquito abundance at lower precipitation levels. However, at higher precipitation values, the trend levelled off. *Anopheles* mosquito abundance was low when relative humidity was below 74% (Fig. 5C). It then increased, reaching a peak at approximately 76% humidity, before declining again. However, as humidity increased beyond 80%, *Anopheles* abundance began to increase once more. The relationship between average temperature and a three-dimensional surface plot further illustrates the interaction between average temperature and relative humidity on *Anopheles* mosquito abundance (Fig. 5D). The plot shows that the density of *Anopheles* mosquito was higher when temperatures ranged between 26–27°C and humidity was between 75 and 80%. However, at extreme values of average temperature and relative humidity (whether too high or too low) *Anopheles* mosquito abundance declined.

### Forecasting the abundance of *Anopheles* mosquitoes based on climatic variables

The predictive performance of the model, as shown in Table 3, demonstrates consistently strong results in both in-sample and out-of-sample forecasts. Within the training set, the models had low mean absolute error (MAE), and root mean square error (RMSE) values in each region, accompanied by high correlations between observed and predicted *Anopheles* abundance (0.82 and 0.98, respectively). The performance on the test set was even stronger, showing reduced error metrics and strong correlation coefficients (r = 0.90 for Tanga and r = 0.99 for Unguja). Moreover, consistently low Theil’s U values (< 0.2) across both regions suggest that the model forecasts significantly outperformed naïve benchmark approaches, as values below 1 indicate superior predictive accuracy. Out-of-sample validation employing independent mosquito count data from Unguja demonstrated a similarly strong correlation coefficient of 0.99, highlighting the model’s capacity to accurately capture temporal dynamics beyond its original training data. Although MAE (20.1) and RMSE (23.2) were notably higher than in-sample metrics (possibly due to increased *Anopheles* abundance or shifts in distribution patterns) the Theil’s U value (0.66) remained below the critical threshold [1], indicating that the model retained strong predictive performance under new data.

**Table 3:** Assessment of predictive accuracy for *Anopheles* mosquito abundance models across Tanga and Unguja, based on training, test, and out-of-sample datasets

Metric	Tanga		Unguja		
	Training set	Test set	Training set	Test set	Out of sample
MAE	3.60	1.69	1.56	1.86	20.1
RMSE	4.54	1.79	3.02	2.28	23.2
Correlation (r) (Observed vs Predicted)	0.82	0.90	0.98	0.99	0.99
Theil’s U	0.13	0.10	0.16	0.08	0.66

**Fig. 6:**
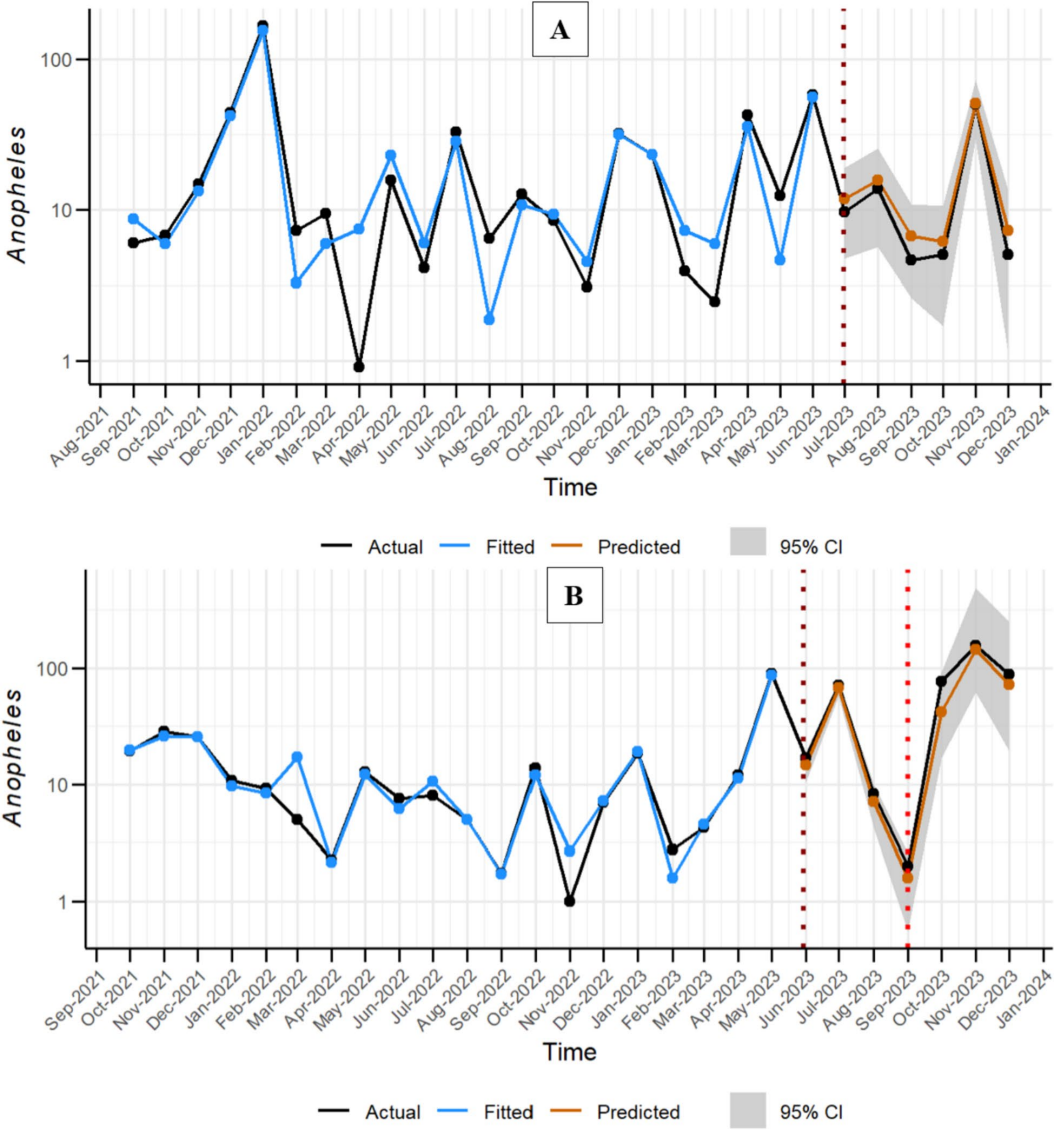
Comparison of observed Anopheles mosquito counts with model-fitted and predicted values for Tanga (**A**) and Unguja (**B**) regions. In Unguja, out-of-sample data from October to December 2023 were also evaluated against model forecasts. The shaded area represents the 95% confidence interval, illustrating the uncertainty around the predicted estimates

Figure 6 depicts the relationship between the predicted and forecasted *Anopheles* mosquito abundance compared to the observed values in both Tanga (Fig. 6A) and Unguja Fig. 6B. During the training period (Tanga: September 2021–July 2023, Unguja: October 2021–June 2023), the predicted values (shown in blue) closely aligned with the observed mosquito abundance (represented in black), demonstrating the model’s ability to capture underlying trends and seasonal patterns. Furthermore, the forecasted values (shown in orange) matched well with the observed data, highlighting the model’s ability to generalize to new datasets. This strong alignment underscores the model's accuracy in fitting historical data and predicting future trends. In Unguja, the model performed exceptionally well on out-of-sampled data, with forecasted values from October to December 2023 closely mirroring the observed mosquito abundance. Remarkably, the model successfully identified the month with the highest number of *Anopheles* mosquitoes (Nov 2023), indicating an outbreak. The model demonstrated strong performance at both the site and shehia levels, as illustrated in S1 Fig 1 and S1 Fig 2. Its accuracy was particularly notable in areas with the highest mosquito abundance and specifically for Unguja, the model effectively predicted increased *Anopheles* populations in Mwera for November 2023, aligning closely with the out-of-sample data.

## Discussion

Malaria transmission is fundamentally driven by the presence and abundance of its primary vectors, *Anopheles* mosquitoes, whose life cycles and population dynamics are strongly moulded by environmental and climatic conditions. Key variables such as temperature, precipitation, and relative humidity influence not only mosquito survival and reproduction but also the rate of *Plasmodium* parasite development within the vector. Consequently, fluctuations in these climatic factors critically determine the risk and intensity of malaria outbreaks. In recognition of these dependencies, increasing attention has been directed toward predictive frameworks that integrate entomological and climatic data to support climate-sensitive early warning systems [57]. These systems are particularly vital in this era of climate change, which is shifting transmission dynamics and expanding the ecological range of malaria vectors. In this study, Generalized Additive Mixed Effects Models (GAMMs) were employed to analyse seasonal patterns of *Anopheles* abundance in relation to climatic variables across the two regions of the United Republic of Tanzania: Tanga in mainland and Unguja in Zanzibar. The models performed strongly both in-sample and out-of-sample, showing low mean absolute error (MAE), low root mean square error (RMSE), Theil’s U values below 1, and high correlation between observed and predicted values (r > 0.8).

The ability of the model to anticipate increases in *Anopheles* mosquito abundance ahead of transmission peaks presents a strategic opportunity for timely public health responses. Such foresight allows for earlier deployment of interventions, including larval habitat management, source reduction, and targeted community engagement. Evidence from previous studies has shown that elevated mosquito abundance is consistently associated with increased malaria risk [58–60]. However, while increasing mosquito populations signal elevated transmission potential, actual outbreak occurrence depends on additional factors such as parasite prevalence, host immunity, and the effectiveness of ongoing interventions. For this reason, peaks in mosquito abundance should be interpreted as early warning indicators rather than deterministic forecasts of malaria epidemics. Nevertheless, the model applied in this study could have detected the surge in *Anopheles* populations that preceded the malaria increase observed in Zanzibar during late 2023 and early 2024, when more than 3500 cases were reported in Unguja and Pemba within just two weeks in January 2024 [6, 7]. Early detection of such vector proliferation through predictive modelling could have supported the timely implementation of WHO-recommended measures, including targeted vector control, environmental management, and intensified community sensitization.

While the model demonstrated strong predictive power in forecasting mosquito abundance and associated malaria risk, its strength lies in capturing the complex and context-specific influence of climatic factors. To better understand these mechanisms, the study examined how temperature, precipitation, and relative humidity shape *Anopheles* dynamics across regions and time. The results revealed region-specific and time-lagged effect. Several studies [19, 23, 61] have highlighted how varying levels of precipitation can create favourable breeding conditions for mosquitoes, thereby increasing their abundance. Our study corroborates this idea, showing a positive and significant relationship between total precipitation and *Anopheles* density for the month of mosquito sampling in Tanga. However, the relationship between precipitation and *Anopheles* density is more complex when considering lagged effects. In Tanga, *Anopheles* abundance decreased following lower precipitation levels lagged by two months, while higher precipitation approximately 200 mm in the current month and 400 mm at a two-month lag, was associated with increased mosquito abundance. This pattern suggests that while heavy rainfall may initially disrupt breeding sites and wash away larvae, it ultimately contributes to the creation of favourable conditions for egg laying and larval development as the water settles. In contrast, the relationship in Unguja was somewhat different. A two-month lag in precipitation showed that lower precipitation increased *Anopheles* density, but once precipitation exceeded a certain threshold of approximately 400 mm, the density of mosquitoes began to decrease. This finding suggests that in Unguja, excess rainfall beyond a certain point might flood breeding sites or alter conditions in a way that becomes less conducive to mosquito survival. These findings are consistent with other studies, such as the one by Walker et al. [62] in Kenya, which also reported a significant association between *Anopheles* density and rainfall with a 1–2-month lag.

Temperature is an important climatic factor that influences *Anopheles* mosquito population dynamics, as it directly affects their survival, development, host-seeking behaviour, and the development of parasite within the mosquito [63]. The impact of temperature on *Anopheles* density in Tanga and Unguja, revealing distinct patterns. In Tanga, both maximum and minimum temperatures showed a linear effect on *Anopheles* abundance. Higher maximum temperatures were associated with an increased density of *Anopheles* mosquitoes, while lower minimum temperatures had the opposite effect, suggesting that warmer conditions support mosquito proliferation, whereas cooler temperatures may limit their survival and activity. These findings are consistent with other studies [23, 61, 63], which reported that higher temperatures favour higher transition rates between mosquito stages, thereby encouraging higher mosquito density. In Unguja, average temperature exhibited a non-linear relationship with *Anopheles* abundance. *Anopheles* density increased when temperatures ranged between 25.5 °C and 27.5 °C but beyond this range the abundance declined. This aligns with previous findings [64], which demonstrated that optimal temperature thresholds promote mosquito development, while excessively high temperatures negatively impact survival rates. Furthermore, temperature interacts with other climatic variables such as humidity and precipitation. The interaction between average temperature and relative humidity in Unguja indicated that moderate temperatures (around 25.5 °C–27.5 °C) and moderate relative humidity (between 76 and 78%) resulted in the highest density of *Anopheles* mosquitoes, while extreme conditions led to a decline.

Relative humidity is another key factor influencing the survival, behaviour, and activity of mosquitoes. Brown et al. [22] highlight that humidity levels can significantly impact mosquito longevity and movement. Our study revealed a complex, non-linear relationship between relative humidity and *Anopheles* mosquito density in both regions. Specifically, when relative humidity levels exceeded 80% in both regions, *Anopheles* abundance increased, likely because mosquitoes become more active and thrive in higher humidity conditions. These findings are supported by previous research, which demonstrated that increased humidity fosters optimal conditions for mosquito proliferation. In particular, Munhenga et al. [65] reported that humidity levels around 85% were most conducive to mosquito collections in northern Kruger National Park, South Africa. The researchers concluded that higher humidity provides a more favourable environment for mosquito survival and activity, ultimately influencing their abundance and distribution. The slightly lower threshold observed in our Tanzanian study (80%) may reflect regional climatic differences, including baseline humidity levels, vegetation structure, and species composition. This highlights the importance of locally calibrated environmental thresholds when interpreting mosquito population dynamics.

Despite its valuable contributions, this study has several limitations. Although it incorporates key weather variables such as temperature, precipitation, and humidity, other important non‑climatic drivers, including land‑use patterns, socio‑economic conditions, vector control interventions, and insecticide resistance, also influence mosquito abundance but were not fully captured in the analysis. Their omission introduces the potential for residual confounding, which may affect the estimated strength of climatic associations. Furthermore, the study focuses on weather‑driven mosquito abundance without integrating real‑time malaria incidence data. Linking mosquito population dynamics with actual disease outcomes would provide a more comprehensive assessment of public health risk and intervention effectiveness. Another limitation relates to species composition. While *Anopheles gambiae* was the dominant vector group and *An. funestus* was detected only in very small numbers, these species occupy distinct ecological niches and respond differently to weather and climatic conditions. By analysing mosquito abundance as a single aggregated outcome, the study may overlook species‑specific climatic sensitivities and introduce bias in interpreting environmental drivers of vector dynamics. Future work should document species composition more explicitly and, where sample sizes permit, analyse species‑specific responses to enhance ecological accuracy and public health relevance. Addressing these limitations will require more comprehensive datasets that integrate ecological, socio‑economic, and operational factors, incorporate real‑time disease surveillance, and capture species‑level variation. Such efforts would improve the accuracy, interpretability, and practical applicability of climate‑informed mosquito control strategies.

## Conclusions

Using the spatio-temporal Generalized Additive Mixed Effects Models (GAMMs), we identified region specific and time lagged effects of climatic variables on *Anopheles* abundance across Tanga and Unguja, with strong predictive performance evidenced by low error metrics and high correlations between observed and predicted values. Despite some limitations, the model offers a scalable, climate sensitive framework to support adaptive surveillance and proactive malaria control strategies, particularly in the context of shifting transmission patterns driven by climate change.

## Supplementary Information

Supplementary Material 1

## Data Availability

The datasets used and/or analysed during the current study are available from the corresponding author on reasonable request.

## Abbreviations

AIC: Akaike information criterion
BIC: Bayesian information criterion
CDC: Centres for disease control and prevention
GAMM: Generalized additive mixed effect model
GCV: Generalized cross-validation
GPM: Global precipitation measurement
IMERG: Integrated multisatellite retrievals for GPM
MAE: Mean absolute error
POWER: Prediction of worldwide energy resources
QAIC: Quasi-Akaike information criterion
RMSE: Root mean square error
VIF: Variance inflation factor
WHO: World Health Organization
