# Supplementary material for: Spatio-temporal modelling and prediction of Anopheles mosquito abundance in Tanga and Unguja, Tanzania: climatic drivers and insights for malaria early warning and vector control strategies
Source: Malar J. 2026 Feb 20;25:145. doi: 10.1186/s12936-026-05798-z (PMC13032517; doi:10.1186/s12936-026-05798-z)
Supplement: Supplementary file 1 — Supplementary Material 1 [file 12936_2026_5798_MOESM1_ESM.docx]

# Supplementary materials


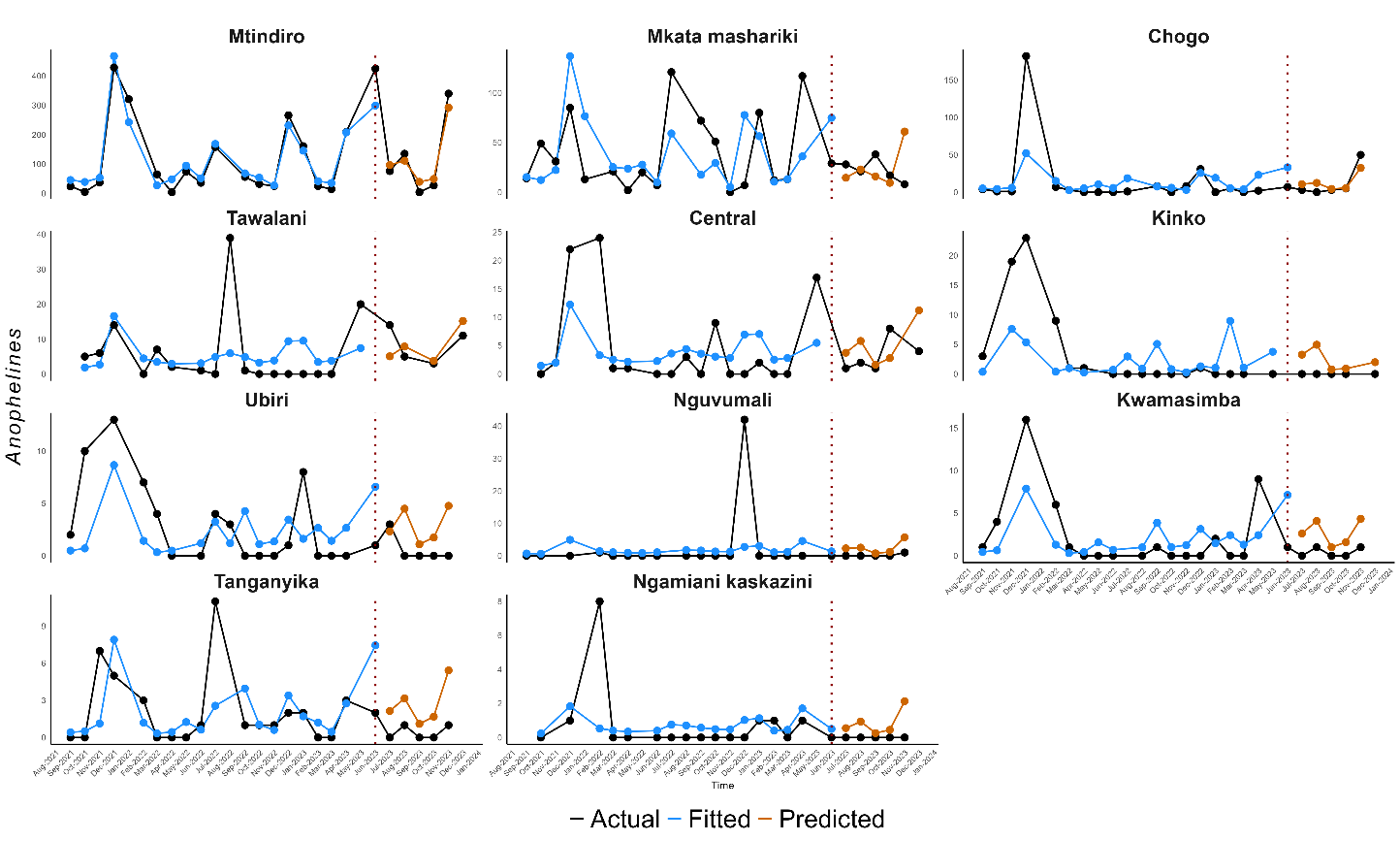


**S1 Fig 1.** The agreements between the observed number of Anopheles mosquitoes versus the fitted valued and predicted values by the model from each site of Tanga.


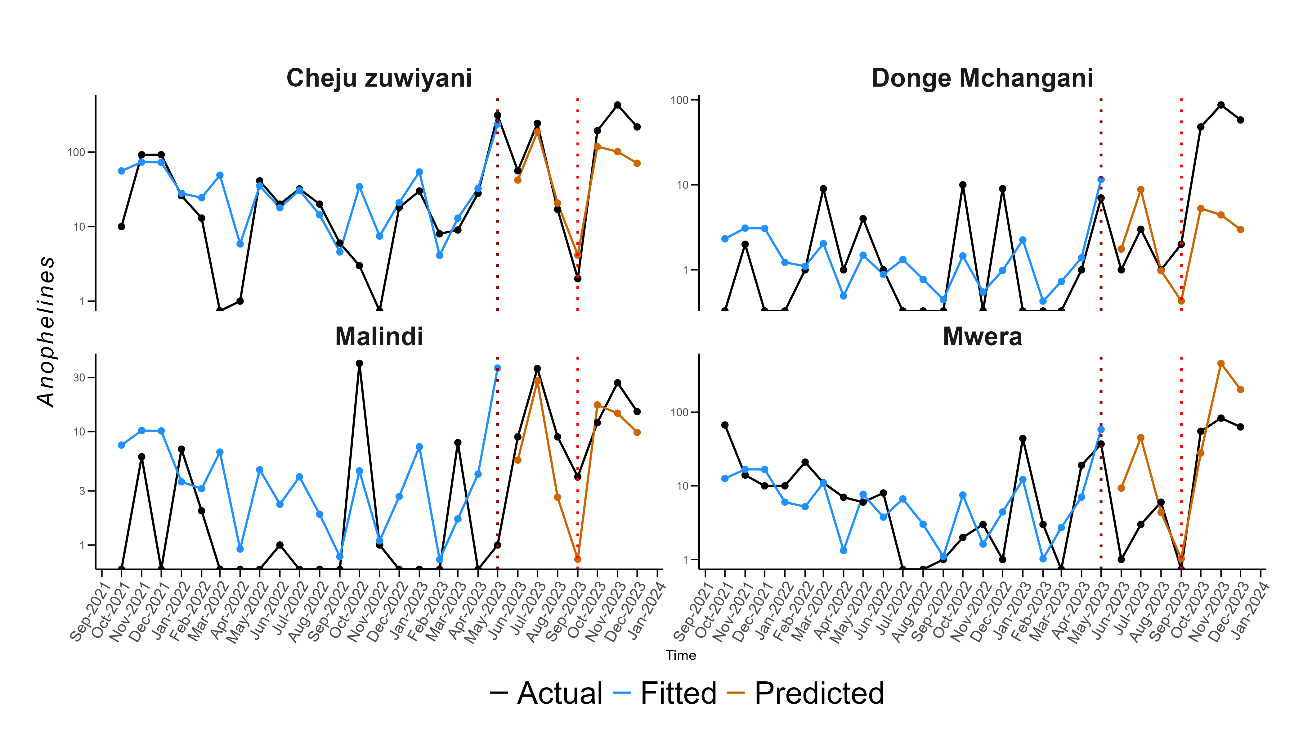


**S1 Fig 2.** The agreement between observed and fitted values, as well as predicted values, for Anopheles mosquito abundance in each shehia of Unguja. Out-of-sample data from October to December 2023 were compared with forecasted values. The model predicted an outbreak of Anopheles mosquitoes in November 2023 in Mwera, which somewhat aligned with the observed data.
